# Cinnamaldehyde Ameliorates Cadmium-Inhibited Root Elongation in Tobacco Seedlings via Decreasing Endogenous Hydrogen Sulfide Production

**DOI:** 10.3390/molecules22010015

**Published:** 2016-12-24

**Authors:** Xie-Feng Ye, Yanfeng Xue, Tianxiao Ling, Yong Wang, Xiao-Na Yu, Changxin Cheng, Guosheng Feng, Liangbin Hu, Zhiqi Shi, Jian Chen

**Affiliations:** 1 Tobacco Science College/National Tobacco Cultivation and Physiology and Biochemistry Research Centre/Key Laboratory for Tobacco Cultivation of Tobacco Industry, Henan Agricultural University, Zhengzhou 450002, China; yexiefeng@163.com (X.-F.Y.); 18837103304@163.com (T.L.); yuxiaona1129@163.com (X.-N.Y.); 2 Nanjing Yangzi Modern Agriculture Investment and Development Co. Ltd., Nanjing 211899, China; hnndxyf@163.com; 3 Chongqing Tobacco Corporation, Chongqing 400023, China; wangyong08326488@163.com; 4 Hongyun Honghe Tobacco Group Co. Ltd., Kunming 650231, China; ccxin1003@163.com; 5 Henan Tobacco Corporation Queshan Branch, Queshan 463200, China; fenggsheng@163.com; 6 Department of Food Science, Henan Institute of Science and Technology, Xinxiang 453003, China; hulb973@163.com; 7 Institute of Food Quality and Safety, Jiangsu Academy of Agricultural Sciences, Nanjing 210014, China; 8 Key Lab of Food Quality and Safety of Jiangsu Province-State Key Laboratory Breeding Base, Nanjing 210014, China

**Keywords:** cinnamaldehyde, hydrogen sulfide, reactive oxygen species, cell death, cadmium, tobacco

## Abstract

Cinnamaldehyde (CA) is natural plant-derived compound that has been highly appreciated for its medicinal properties. However, little information is known about the regulation of plant intrinsic physiology by CA. To address these gaps, physiological, histochemical, and biochemical approaches were applied to investigate CA-facilitated cadmium (Cd) tolerance in the roots of tobacco (*Nicotiana tabacum*) seedlings. Treatment with CdCl_2_ at 20 μM for 72 h resulted in the significant decrease in root elongation by 40.39% as compared to control. CA alleviated Cd-inhibited root elongation in dose- and time-dependent manners. The addition of CA at 20 μM induced significant increase in root elongation by 42.58% as compared to Cd treatment alone. CA abolished Cd-induced ROS (reactive oxygen species) accumulation, lipid peroxidation, loss of membrane integrity, cell death, and free Cd^2+^ accumulation in roots. CA blocked the Cd-induced increase in the endogenous H_2_S level through the down-regulation of *d-cysteine desulfhydrase* (*DCD*) expression. H_2_S scavenger hypotaurine (HT) or potent H_2_S-biosynthetic inhibitor dl-propargylglicine (PAG) were able mimic the action of CA on the blockade of Cd-induced H_2_S accumulation, cell death, and growth inhibition. Enhancement of the endogenous H_2_S level with NaHS (H_2_S donor) abrogated all the beneficial capabilities of CA, HT, and PAG. Collectively, these results suggest that CA has great potential to confer plant tolerance against Cd stress, which is closely associated with its capability to inhibit Cd-induced H_2_S production. This study not only provides evidences for the regulation of plant physiology by CA but also sheds new light on the cross-talk between CA and H_2_S in physiological modulations.

## 1. Introduction

Cinnamaldehyde (CA) is an important component of essential oil obtained from cinnamon trees and other *Cinnamomum* species [1,2]. CA is a bioactive compound that has been highly appreciated for its medicinal properties, such as anti-oxidative and anti-inflammatory properties [3,4], anti-microbial activity [5], anti-viral activity [6], and anti-cancer activity [7]. The clinical relevance of CA has been closely linked to the regulation of immune pathways in mammalian cells, which can be mediated by several important signaling molecules, such as Ca^2+^ and nitric oxide (NO) [8,9]. CA also shows great potential to maintain food quality due to its anti-microbial activity [10]. The bioactivity of CA on both mammals and microorganisms has been identified extensively, but little information is known about the regulation of plant-resistant physiology by CA.

Plant growth is impacted by multiple environmental stresses, including heavy metals. Cadmium (Cd) pollution is becoming a global problem because of the natural and anthropogenic release of Cd into the environment [11]. Ionic cadmium (Cd^2+^) is the ubiquitous form of Cd existing in the environment. Cd^2+^ can be easily absorbed and accumulated in plants, which further shows adverse effects on plant growth and poses a risk to human health through the food chain [12]. Cd stress frequently induces the accumulation of ROS (reactive oxygen species), leading to the occurrence of oxidative stress, cell death, and growth inhibition in plants [13].

Hydrogen sulfide (H_2_S) is a toxic gaseous molecule in the environment. However, H_2_S is also an endogenously-generated regulator of a variety of biological processes in mammals and plants [14,15]. H_2_S and CA share many downstream regulatory networks that involve ROS, NO, and Ca^2+^ in mammalian cells [8,16,17], but the interaction between H_2_S and CA during physiological modulation is little known. In mammals, pyridoxal 5′-phosphate (PLP)-dependent family proteins, cystathionine-γ-lyase (CSE) and cystathionine-β-synthase (CBS), are two key enzyme for the endogenous production of H_2_S [18]. In plant cells, H_2_S can be endogenously produced from cysteine desulfuration catalyzed by l-cysteine desulfhydrase (LCD) and d-cysteine desulfhydrase (DCD), also belonging to PLP-dependent protein family [19,20]. H_2_S has been considered as an important regulator for the modulation of plant physiology under various environmental stimuli, including heavy metal stress [21]. It has been documented that H_2_S is involved in the regulation of plant responses to Cd stress [22,23,24,25,26,27,28]. The interaction between H_2_S and other signaling molecules (e.g., NO and Ca^2+^) to modulate ROS homeostasis has been identified in various plant species under heavy metal stress [24,26,29]. Thus, it can be speculated that the interaction between CA and H_2_S probably exists during plant physiological regulation under environmental stimuli.

In this work, we first studied the alleviating effect of CA on CdCl_2_-induced growth inhibition, oxidative injury, and free Cd^2+^ accumulation in the root of tobacco (*Nicotiana tabacum*) seedlings. To further investigate the hypothesis that H_2_S may be involved in CA-mediated alleviation of Cd-induced phytotoxicity, we detected the effect of CA on endogenous H_2_S distribution and the expression level of *LCD* and *DCD* in CdCl_2_-treated roots. Then plant roots were treated with CA, CdCl_2_, the H_2_S donor sodium hydrosulfide (NaHS), the H_2_S scavenger hypotaurine (HT), and potent H_2_S-biosynthetic inhibitor _DL_-propargylglycine (PAG), alone or in various combinations, for the determination of endogenous H_2_S, root elongation, and cell death. Finally, the possible mechanisms for the interaction among these parameters are discussed.

## 2. Results

### 2.1. CA Significantly Mitigated Cd-Induced Inhibition of Root Elongation in Tobacco Seedlings

**Figure 1:**
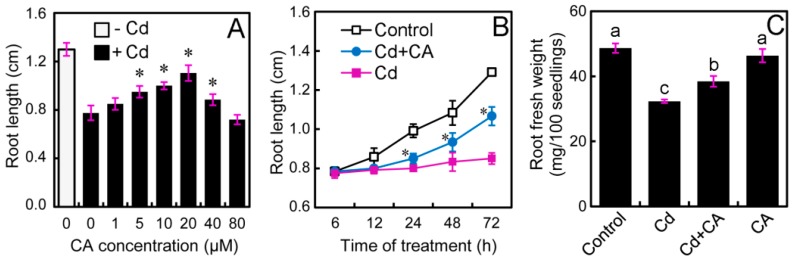
The effect of CA on root growth of tobacco seedlings under Cd stress. (**A**) In the presence of CdCl_2_ at 20 μM, the roots of seedlings were treated with CA at different concentrations (0–80 μM) for 72 h. Then the root length was measured; (**B**) the roots of seedlings were exposed to 20 μM of CdCl_2_ and 20 μM of CA simultaneously for 6, 12, 24, 48, and 72 h, respectively, for the measurement of root length; (**C**) the roots of seedlings were treated with water, 20 μM of CdCl_2_, 20 μM of CA, alone or their combinations for 72 h. Then the roots were harvested for the quantification of fresh weight. The asterisk (*) in (**A**,**B**) indicated that the mean value of six replicates was significantly different between Cd + CA treatment and Cd treatment alone (*p* < 0.05, ANOVA). Different letters in (**C**) indicated that the mean values of three replicates are significantly different between the treatments (*p* < 0.05, ANOVA, LSD).

Treatment with CdCl_2_ at 20 μM for 72 h resulted in the significant decrease in root elongation by 40.39% as compared to control (Figure 1A). CA with different concentrations (0–40 μM) was added to the treatment solution to investigate the effect of CA on the modulation of root elongation under Cd stress. As compared to Cd treatment alone, the addition of CA at 5, 10, 20, and 40 μM, induced significant increase in root elongation by 22.58%, 29.03%, 42.58%, and 14.19%, respectively (Figure 1A). CA at 80 μM failed to stimulate root elongation under Cd exposure (Figure 1A). CA at 20 μM showed the greatest effect on the alleviation of Cd-induced inhibition of root elongation. Therefore, 20 μM of CA was used for further estimation of physiological responses. In a time-course experiment up to 72 h, root elongation began to significantly increase after treatment with CA + Cd for 24 h as compared to Cd treatment alone. CA at 20 μM showed continuous alleviating effect on Cd-induced inhibition of root elongation after treatment for 24–72 h (Figure 1B). In addition, the addition of CA significantly enhanced root fresh weight under Cd stress (Figure 1C). These results suggested that CA recovered root growth of tobacco seedlings from Cd stress.

### 2.2. CA Inhibited Cd-Induced ROS Over-Generation in the Roots of Tobacco Seedlings

**Figure 2:**
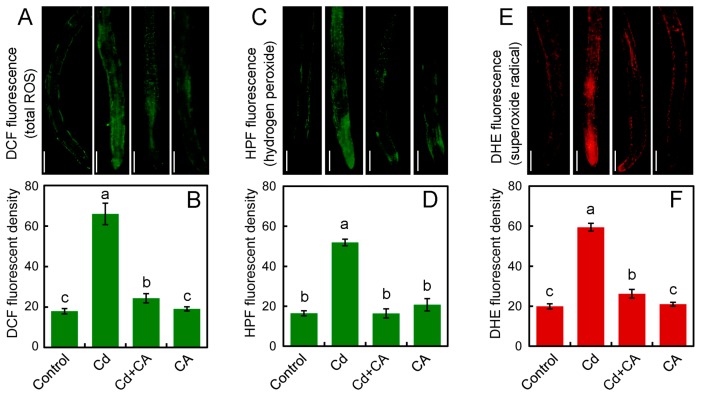
The effect of CA on ROS accumulation in the roots of tobacco seedlings under Cd stress. The roots of seedlings were treated with distilled water (control), CdCl_2_ (20 μM), CdCl_2_ (20 μM) + CA (20 μM), and CA (20 μM) for 72 h. (**A**) The roots were loaded with DCFH-DA for the observation of total ROS fluorescence with fluorescent microscope; (**B**) The DCF fluorescent density was quantified to indicate the relative total ROS level in roots; (**C**) the roots were loaded with HPF for the observation of endogenous H_2_O_2_ with fluorescent microscope; (**D**) the HPF fluorescent density was quantified to indicate the relative H_2_O_2_ level in roots; (**E**) the roots were loaded with DHE for the observation of endogenous O_2_^•^¯ with fluorescent microscope; (**F**) the DHE fluorescent density was quantified to indicate the relative O_2_^•^¯ level in roots. Bar = 0.5 mm. Different letters in (**B**), (**D**), and (**F**) indicate that the mean values of three replicates are significantly different between the treatments (*p* < 0.05, ANOVA, LSD).

Fluorescent probe DCFH-DA (2′,7′-dichlorofluorescein diacetate), reacting specifically with ROS to form DCF (2′,7′-dichlorofluorescein) emitting green fluorescence, was used to detect total ROS in situ in roots. Cd-treated roots showed stronger DCF fluorescence than that of control (Figure 2A). However, Cd + CA treatment weakened DCF fluorescence in roots as compared to Cd treatment alone (Figure 2A). The quantification of DCF fluorescent density suggested that the relative total ROS content in Cd-treated root significantly increased as compared to the control (Figure 2B). Nevertheless, Cd + CA treatment led to remarkable decrease in the relative total ROS content in root by 63.09% as compared to Cd treatment alone (Figure 2B). CA treatment alone did not significantly change total ROS content in roots under normal growth conditions (Figure 2B).

Hydrogen peroxide (H_2_O_2_) and superoxide radical (O_2_^•^¯) are two typical ROS induced by heavy metal stress in plants [30]. Endogenous H_2_O_2_ in roots was detected in situ with the specific fluorescent probe HPF (3′-(*p*-hydroxyphenyl) fluorescein), presenting green fluorescence. As expected, H_2_O_2_ content in roots increased remarkably by 215.40% under Cd exposure, which was decreased to the control level by the addition of CA (Figure 2C,D). Endogenous O_2_^•^¯ in roots was selectively detected in situ with the specific fluorescent probe DHE (dihydroethidium), presenting red fluorescence (Figure 2E). Cd treatment led to a significant increase in O_2_^•^¯ content in roots by 198.07% as compared to control (Figure 2F). However, treatment with Cd + CA significantly decreased O_2_^•^¯ content by 55.91% in roots as compared to Cd treatment alone (Figure 2F). Neither H_2_O_2_ nor O_2_^•^¯ in roots was affected by CA treatment alone (Figure 2D,F). These results suggested that CA was able to inhibit ROS accumulation in the roots of tobacco seedlings under Cd stress.

### 2.3. CA Ameliorated Cd-Induced Oxidative Injury in the Roots of Tobacco Seedlings

**Figure 3:**
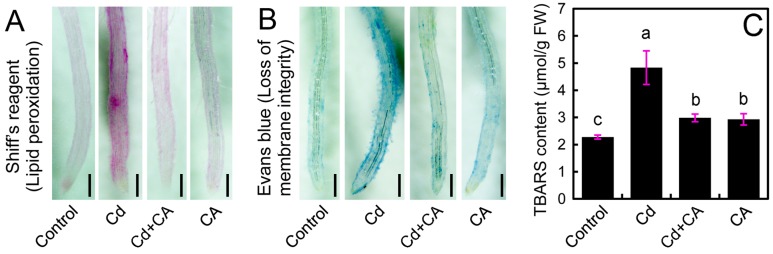
The effect of CA on lipid peroxidation, loss of membrane integrity, and TBARS content in the roots of tobacco seedlings under Cd stress. The roots of seedlings were treated with distilled water (control), CdCl_2_ (20 μM), CdCl_2_ (20 μM) + CA (20 μM), and CA (20 μM) for 72 h. After treatment, the roots were stained with Shiff’s reagent (**A**) and Evans blue (**B**), respectively, followed by photographing with a stereoscopic microscope. Bar = 0.5 mm; and (**C**) the roots after treatment were harvested for the determination of TBARS content. Different letters indicate that the mean values of three replicates are significantly different between the treatments (*p* < 0.05, ANOVA, LSD).

Lipid peroxidation of the cell membrane is one of the typical consequences of oxidative injury in plants in response to abiotic stress [31]. In the present study, peroxidation of membrane lipids and the loss of plasma membrane integrity were detected in vivo by using histochemical staining with Shiff’s reagent and Evans blue, respectively. Cd-treatment alone led to extensive staining in roots, while the roots treated with CA + Cd and control had only light staining (Figure 3A,B). TBARS (thiobarbituric acid reactive substances) content is a typical indicator of lipid peroxidation. Cd treatment induced significant increase in TRARS level by 112.07% in roots as compared to control (Figure 3C). However, Cd + CA treatment led to the significant decrease in TBARS content by 38.26% in roots as compared to the control (Figure 3C). These results suggested that CA remarkably attenuated Cd-induced oxidative injury in the roots of tobacco seedlings.

### 2.4. CA Decreased Free Cd^2+^ Accumulation in the Roots of Tobacco Seedlings

**Figure 4:**
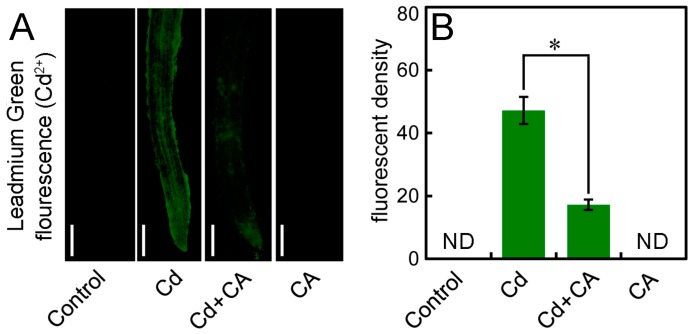
The effect of CA on free Cd^2+^ in the roots of tobacco seedlings under Cd stress. The roots of seedlings were treated with distilled water (control), CdCl_2_ (20 μM), CdCl_2_ (20 μM) + CA (20 μM), and CA (20 μM) for 72 h. (**A**) The roots were loaded with Leadmium^TM^ Green AM and photographed with a fluorescent microscope. Bar = 0.5 mm; (**B**) The Leadmium Green fluorescent density was quantified to indicate the relative Cd^2+^ level in roots. The asterisk (*) indicates that the mean value of three replicates was significantly different between Cd + CA treatment and Cd treatment alone (*p* < 0.05, ANOVA).

Leadmium^TM^ Green AM (Invitrogen Molecular Probes, Inc., Eugene, OR, USA) with the capability of reacting with free Cd^2+^ to emit green fluorescence was used to selectively detect free Cd^2+^ in roots. In the present study, we did not detect any fluorescent signal in the roots of control and CA treatment alone. The roots treated with Cd alone showed much stronger fluorescence than that of Cd + CA treatment (Figure 4A). The fluorescent density of Leadmium Green significantly decreased by 63.56% in roots under Cd + CA treatment as compared to Cd treatment alone (Figure 4B). These results indicated that the addition of CA significantly prohibited free Cd^2+^ accumulation in Cd-treated roots.

### 2.5. CA Remarkably Repressed Cd-Induced Production of Endogenou H_2_S in the Roots of Tobacco Seedlings

**Figure 5:**
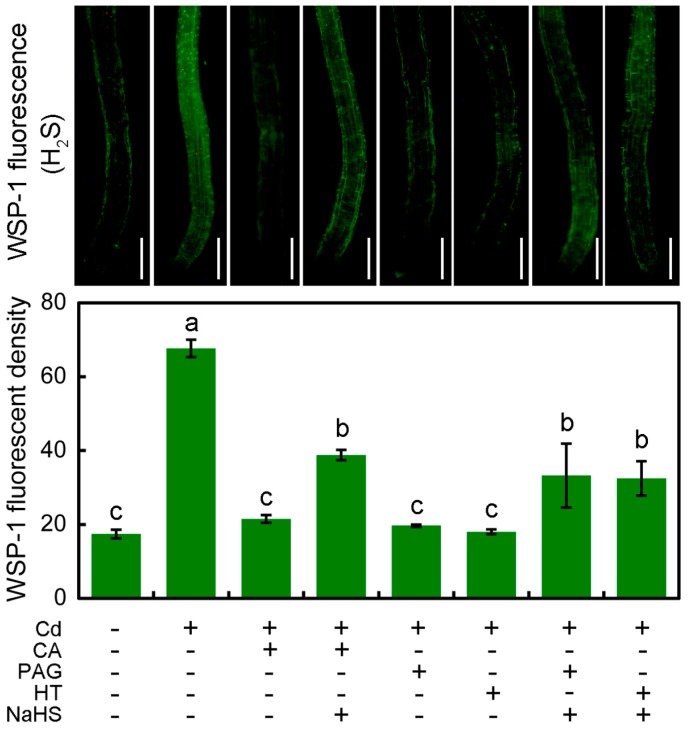
The effect of CA on endogenous H_2_S content in the roots of tobacco seedlings under Cd stress. The roots of seedlings were treated with distilled water (control), CdCl_2_ (20 μM), CA (20 μM), NaHS (20 μM), PAG (2 μM), HT (20 μM), alone or their combinations for 72 h. Then the roots were loaded with WSP-1 for the observation of green fluorescence and the quantification of fluorescent density. Bar = 0.5 mm. Different letters indicated that the mean values of six replicates were significantly different between the treatments (*p* < 0.05, ANOVA, LSD).

Specific molecular probe WSP-1 (Washington State Probe 1) was applied to selectively detect endogenous H_2_S in roots. The endogenous H_2_S level in roots significantly increased by 270.16% in response to Cd stress, which was decreased to the control level by the addition of CA (Figure 5). The addition of NaHS (H_2_S donor) was able to enhance the endogenous H_2_S content in roots treated with Cd + CA (Figure 5). PAG (H_2_S-biosynthetic inhibitor) or HT (H_2_S scavenger) showed similar effects on the endogenous H_2_S content in Cd-treated roots as compared to CA. The application of PAG or HT significantly decreased endogenous H_2_S level in the Cd-treated roots, which could be reversed by the addition of NaHS, as well (Figure 5).

**Figure 6:**
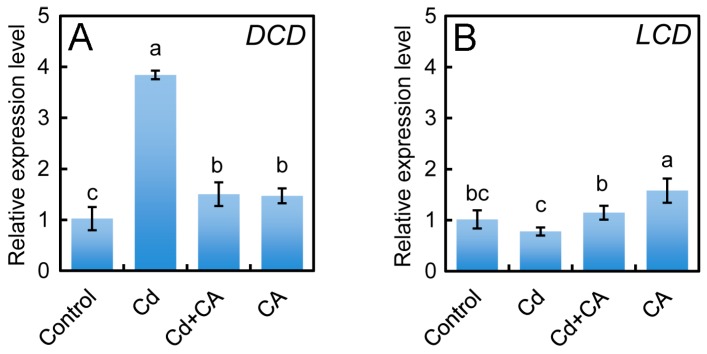
The effect of CA on the expression of *DCD* and *LCD* in the roots of tobacco seedlings under Cd stress. The roots of seedlings were treated with distilled water (control), CdCl_2_ (20 μM), CdCl_2_ (20 μM) + CA (20 μM), and CA (20 μM) for 72 h. Then the roots were harvested for RNA extraction to analyze the expression level of *DCD* (**A**) and *LCD* (**B**). Different letters indicated that the mean values of three replicates were significantly different between the treatments (*p* < 0.05, ANOVA, LSD).

To understand how Cd and CA regulate the production of endogenous H_2_S, the expression level of *LCD* and *DCD* in roots upon different treatments was analyzed by using Real-time RT-PCR (reverse transcription-polymerase chain reaction). The expression level of *DCD* significantly increased by 274.61% in Cd-treated roots compared to control. Treatment with Cd + CA resulted in the dramatic decrease in *DCD* expression level by 60.84% as compared to Cd treatment alone (Figure 6A). However, Cd treatment alone led to a slight decrease in the expression level of *LCD* in roots as compared to control or Cd + CA treatment (Figure 6B). Treatment with CA alone induced a slight increase in the expression level of *DCD* and *LCD* (Figure 6).

### 2.6. CA Alleviated Cd-Inhibited Root Elongation by Modulating Endogenous H_2_S in Tobacco Seedlings

**Figure 7:**
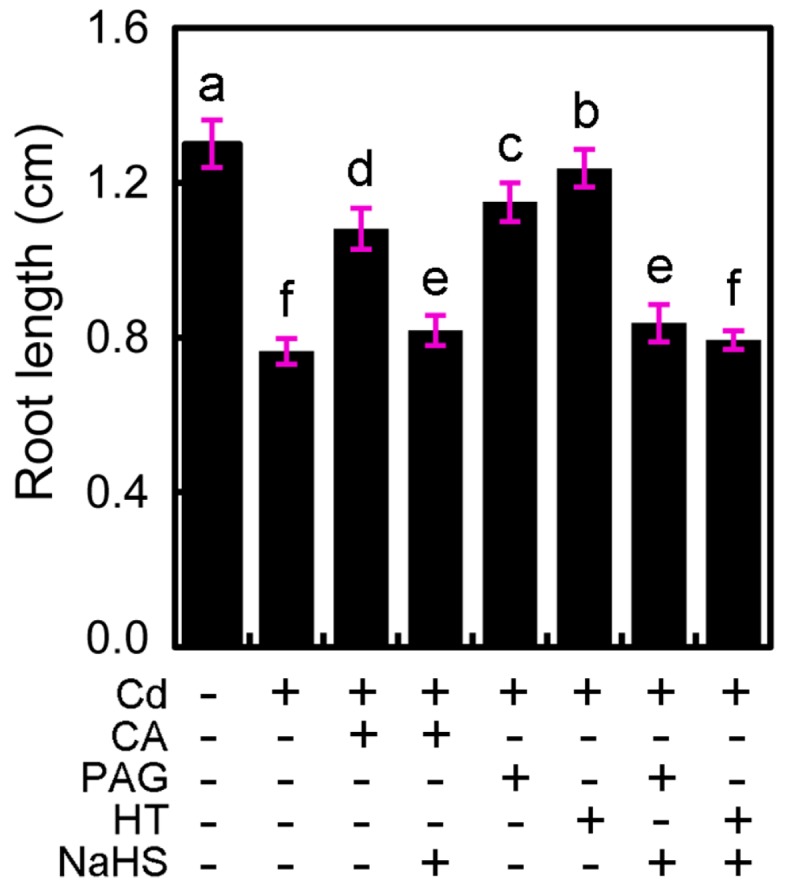
The effect of CA, NaHS, PAG, and HT on root elongation in tobacco seedlings under Cd stress. The roots of seedlings were treated with distilled water (control), CdCl_2_ (20 μM), CA (20 μM), NaHS (20 μM), PAG (2 μM), HT (20 μM), alone or their combinations for 72 h, for the measurement of root length. Different letters indicated that the mean values of five replicates were significantly different between the treatments (*p* < 0.05, ANOVA, LSD).

CA significantly induced the increase in root elongation upon Cd stress, which was diminished by the addition of H_2_S donor NaHS (Figure 7). Similar to CA, treatment with Cd + PAG or Cd + HT induced significant increase in root elongation as compared to Cd treatment alone (Figure 7). The addition of NaHS diminished the promoting effect of PAG or HT on root elongation under Cd stress, as well (Figure 7). These results suggested that endogenous H_2_S played negative role in the regulation of root elongation under Cd stress, and CA was able to alleviate Cd-inhibited root growth by decreasing the endogenous H_2_S in tobacco seedlings.

### 2.7. CA Decreased Cd-Inhibited Cell Death by Modulating Endogenous H_2_S in the Root of Tobacco Seedlings

**Figure 8:**
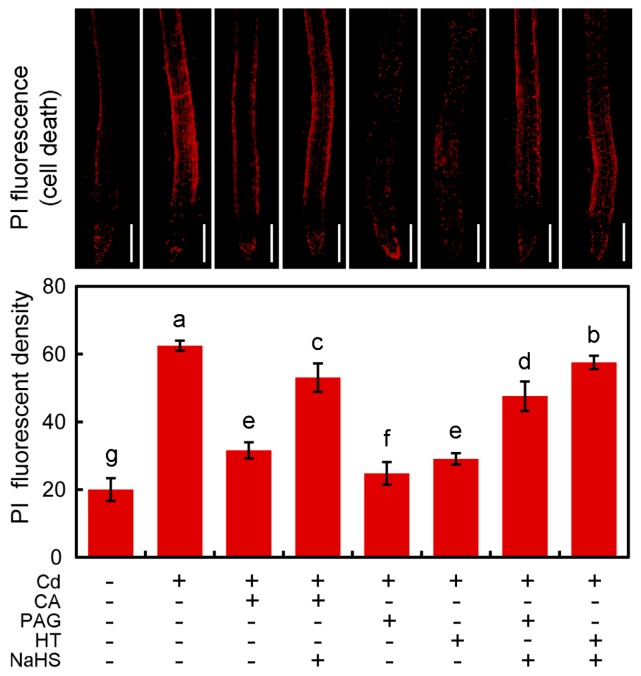
The effect of CA, NaHS, PAG, and HT on cell death in the root of tobacco seedlings under Cd stress. The roots of seedlings were treated with distilled water (control), CdCl_2_ (20 μM), CA (20 μM), NaHS (20 μM), PAG (2 μM), HT (20 μM), alone or their combinations for 72 h. Then the roots were loaded with PI the microscopic observation of red fluorescence and the quantification of fluorescent density. Bar = 0.5 mm. Different letters indicated that the mean values of three replicates were significantly different between the treatments (*p* < 0.05, ANOVA, LSD).

PI (propidium iodide) staining was performed to indicate cell death in the roots of tobacco seedlings. Cd treatment alone resulted in significant increase in PI fluorescent density by 216.95% as compared to control, suggesting that Cd stress induced severe cell death in roots (Figure 8). Treatment with Cd + CA resulted in the significant decrease in PI fluorescent density by 50.41% as compared to Cd treatment alone. Similarly, the addition of PAG or HT was able to remarkably decrease PI fluorescent density in Cd-treated roots (Figure 8). However, the addition of NaHS diminished the effect of CA, PAG, or HT on PI fluorescent density in Cd-treated roots (Figure 8). These results suggested that endogenous H_2_S played positive role in triggering cell death in Cd-treated root, and CA was able to decrease Cd-induced cell death by decreasing the endogenous H_2_S in tobacco seedlings.

### 2.8. Hierarchical Cluster Analysis of the Interaction of CA and H_2_S in Roots Exposed to Cd

**Figure 9:**
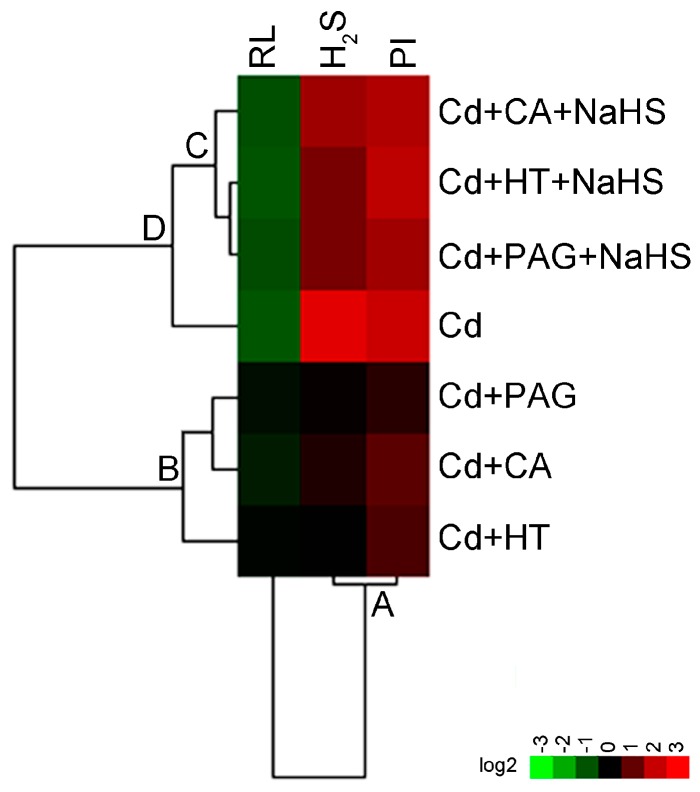
Hierarchical cluster analysis of interaction between CA and H_2_S on physiological responses of tobacco seedling roots under Cd stress. The relative data of RL (root length), H_2_S content (indicated by WSP-1 fluorescent density), and cell death (indicated by PI fluorescent density) in roots with different treatment, were selected for cluster analysis. All of the data were presented as relative fold changes with respect to control. The cluster color bar was shown as a log_2_ fold change. Red indicates an increase while green indicates a decrease.

Based on the obtained data of RL (root length), endogenous H_2_S content (indicated by WSP-1 fluorescent density), and cell death (indicated by PI fluorescent density) in roots upon different treatments (Figure 5, Figure 7, and Figure 8), hierarchical clustering was performed to analyze the relationship among biochemical parameters or different treatments (Figure 9). For the biochemical parameters, H_2_S and PI were in the same group (Figure 9, A), indicating that they were negatively related to root elongation upon different treatments. For different treatments, Cd + CA, Cd + PAG, and Cd + HT were in the same group (Figure 9, B). CA, PAG, and HT showed similar effects on the decrease in Cd-induced H_2_S in roots, which was associated with the recovery of root from Cd-induced growth inhibition and cell death (Figure 9, B). The addition of NaHS was able to block the effect of PAG, HT, or CA on all the parameters in Cd-roots (Figure 9, C), showing similar patterns with Cd-treatment alone (Figure 9, D).

## 3. Discussion

The medicinal properties of CA have been reported extensively [2], but the role of CA in the regulation of plant physiology remains elusive. It has been demonstrated that CA is capable of protecting mammalian cells against stress conditions [32,33]. In the present study, four lines of evidence indicate that CA is able to protect tobacco seedlings from Cd stress by decreasing the endogenous H_2_S in roots. Firstly, CA attenuated Cd-induced growth inhibition, oxidative injury, and cell death in roots. Secondly, Cd stimulated *DCD*-dependent H_2_S generation in roots, which was repressed by CA. Thirdly, the application of the H_2_S-biosynthetic inhibitor (PAG) of the H_2_S scavenger (HT) showed similar action to CA. PAG or HT blocked the increase in the endogenous H_2_S level in Cd-treated roots, coinciding with the recovery of root growth and the decrease in cell death under Cd stress. Fourthly, the abilities of CA, HT, or PAG to alleviate Cd-induced phytotoxicity were greatly reduced by enhancing the endogenous H_2_S with NaHS.

Growth inhibition is a typical symptom in plants in response to organic and inorganic contaminants [34,35]. Measurement of root elongation has been recommended as a reliable method for phytotoxicity testing of hazardous substances, including heavy metals [35,36]. Our current results suggested that CA at a proper dose significantly attenuated Cd-induced inhibition of root elongation, suggesting that CA protected plants from Cd toxicity. However, CA at high dose (40 μM) showed decreased ability of inducing plant tolerance against Cd as compared to 20 μM of CA. CA at 80 μM even failed to restore root elongation under Cd exposure. It has been demonstrated that CA shows adverse effects on plant growth [37]. We also observed that treatment with CA at high dose alone was able to inhibit root elongation (data not shown), which may compromise its protective effect against Cd stress. The antioxidative and antiperoxidative properties of CA are important for its capacity protecting mammalian cells from stress conditions [38]. Here we found that CA combated Cd toxicity in plants through the similar mechanism. Cd-induced phytotoxicity is largely attributed to ROS accumulation in plant cells [39,40]. The over-generated ROS always triggers oxidative injury and cell death in plants [41]. ROS frequently attack membrane lipids to induce lipid peroxidation of the plasma membrane indicated by TBARS [42,43]. In the present study, CA attenuated Cd-induced lipid peroxidation and plasma membrane damage, which may have resulted from the repression of ROS accumulation (including H_2_O_2_ and O_2_^•^¯) in the roots of tobacco seedlings upon Cd exposure. CA has the ability to scavenge ROS directly in vitro [38], which may partially contribute to the alleviation of Cd-induced cytotoxicity in the roots of tobacco seedlings. NADPH (nicotinamide adenine dinucleotide phosphate) oxidase located in the plasma membrane has been suggested to be a major source for ROS generation in plants under environmental stimuli [44]. Cd-induced tobacco cell death is ascribed to NADPH oxidase-dependent ROS production [39,45]. In mammalian cells, the cardioprotective effect of CA has been linked to its ability to decrease NADPH activity and ROS production [46]. In Cd-treated tobacco roots, whether CA inhibits ROS accumulation through the similar mechanism needs to be studied further. Moreover, the ability of CA to decrease ROS in vivo has been closely related to the regulation of various intracellular signaling in mammalian cells [46,47,48]. Notably, the inhibition of endogenous NO generation is critical for the anti-inflammatory and anti-carcinogenic activity of CA [4,49,50]. The interaction between NO and ROS is important for triggering cell death in both mammals and plants [51,52]. It has been reported that NO-dependent H_2_O_2_ production is required for Cd-induced cell death in *Arabidopsis* suspension cells [53]. Therefore, whether CA prevents Cd-induced cell death by inhibiting NO/H_2_O_2_ interaction in tobacco seedlings remains to be investigated further.

The knowledge about H_2_S in plants are relatively limited as compared to those in mammals [15]. Recently, H_2_S has been drawing great attention because of its emerging role in the regulation of plant physiology [21]. The increase in endogenous H_2_S level has been observed in various plant species upon Cd exposure, but the physiological role of the suppression of Cd-induced H_2_S was rarely concerned [24,25,26,27,28]. In the present study, Cd stress stimulated the expression of *DCD* and the production of endogenous H_2_S in roots, whereas blockade H_2_S accumulation (back to normal levels) with PAG or HT led to the alleviation of Cd-induced phytotoxicity. These results suggested that endogenously-generated H_2_S mediated Cd toxicity in tobacco seedlings. It has been documented that H_2_S promotes NADPH oxidase-dependent H_2_O_2_ production in *Arabidopsis* roots under salt stress [54]. Therefore, CA-blocked H_2_S production may partially contribute to the inhibitory effect of CA on H_2_O_2_ accumulation in Cd-treated roots.

H_2_S is a kind of “double-edged sword” during the disease responses in mammals. In addition to the protective role of H_2_S against cell dysfunction [14], endogenous H_2_S can also act as an important physiological mediator in inflammation of mammalian cells [55,56,57]. During the inflammation process, the expression of *CSE* is stimulated leading to the increase in H_2_S biosynthesis. However, the inhibition of H_2_S production with PAG shows anti-inflammatory activity [55,56,57]. These studies are similar to our current results that DCD-dependent H_2_S formation contributed to Cd-induced toxicity in tobacco roots. CA significantly repressed the expression of *DCD* and the production of endogenous H_2_S in Cd-treated roots, leading to the alleviation of Cd-induced phytotoxicity in tobacco seedlings. In addition, we found that the elevation of endogenous H_2_S triggered cell death in Cd-treated roots, which could be blocked by CA. It has been suggested that H_2_S induces caspase-dependent cell death in human cells [58,59]. Caspase-dependent cell death has been found in plants under Cd stress [60]. Thus, it is of interest to further investigate whether CA regulates caspase-dependent cell death by modulating endogenous H_2_S in tobacco seedlings under Cd stress.

The meristem zone (MZ) in the root apex is vital for root elongation. MZ activity is regulated by a complex signaling network, involving ROS, abscisic acid, auxin, and cytokinin, etc. [61,62]. It has been reported that Cd inhibits root meristem growth by repressing NO-mediated auxin signaling in *Arabidopsis* [63]. ROS play important roles in the regulation of by regulating cell proliferation and differentiation [62,64]. Here we found that CA significantly suppressed ROS accumulation in the root apex under Cd exposure. Interestingly, CA is capable of modulating cell proliferation and differentiation in mammals [65]. Therefore, it is of interest to further investigate whether CA-suppressed ROS accumulation modulates apical meristem activity in the root tip of tobacco seedlings under Cd exposure.

As compared to the medical properties of CA, little information is known about the regulation of plant physiology by CA. Our current study proposes that CA is capable of conferring plant tolerance against Cd stress. CA significantly attenuates Cd-inhibited root growth in tobacco seedlings by blocking Cd-induced oxidative injury and cell death. Generally, natural compounds tend to combat stress-induced oxidative injury by enhancing anti-oxidative capacity to remove over-generated ROS in mammalian cells [66]. However, our current study suggests that CA seems to inhibit the production of ROS, which is mediated by endogenous H_2_S in tobacco seedlings under Cd exposure. The detailed mechanism for CA-facilitated plant adaption to Cd is still elusive, but our current results provide evidences for the regulation of plant-resistant physiology by CA. Therefore, CA would have great potential to be applied to help plants grow in heavy metal-polluted environments. Such findings could extend our knowledge of CA and H_2_S in the regulation of plant physiology, which is vital for both fundamental and applied plant biology.

## 4. Materials and Methods

### 4.1. Plant Culture, Treatment, and Chemicals

Seeds of *Nicotiana tabacum* were germinated for 24 h in the dark on wet filter paper in a Petri dish at 25 °C. Then 30 selected identical seedlings with 0.5 cm radicles were transferred to another wet filter paper in a new Petri dish. Then different treatment solutions (5 mL) were added into the Petri dish. Seedling roots were immersed into the treatment solution for different treatment times. Petri dishes with seedlings were put in a photosynthetic active radiation chamber of 200 μmol/m^2^/s, a photoperiod of 12 h, and a temperature of 25 °C. After treatment, the roots were harvested and washed with distilled water for histochemical, physiological, and biochemical analyses.

According to different experimental designs, the roots of seedlings were exposed to CdCl_2_ (20 μM), CA (1–80 μM), NaHS (20 μM), PAG (2 μM), and HT (20 μM), alone or in combined solutions for various treatment times (0–72 h). NaHS, PAG, and HT were applied as an H_2_S donor, H_2_S biosynthesis inhibitor, and H_2_S scavenger, respectively [67]. All the chemicals used in this study were analytical reagents obtained from Sigma-Aldrich (St. Louis, MO, USA).

### 4.2. Histochemical Analysis of Total ROS in Roots

Intracellular total ROS in roots was visualized using specific fluorescent probe DCFH-DA in situ described by Foreman et al. [68]. The roots of seedlings were incubated in 10 μM of DCFH-DA at 25 °C for 10 min. Then the roots were rinsed with distilled water three times, followed by visualization (excitation 488 nm and emission 525 nm) with a fluorescence microscope (ECLIPSE, TE2000-S, Nikon, Melville, NY, USA). The relative fluorescent density of DCF was analyzed using Image-Pro Plus 6.0 (Media Cybernetics, Inc., Rockville, MD, USA). The roots without incubated with DCFH-DA were detected under the same conditions to exclude the possibility for the autofluorescence of root tissues. A similar procedure was also performed before using other fluorescent probes in this study.

### 4.3. Histochemical Detection of Intracellular H_2_O_2_ in Roots

Intracellular H_2_O_2_ in roots was visualized in situ using specific fluorescent probe HPF described by Dunand and Crevecoeur [69]. The roots of seedlings after treatment were incubated in 5 μM of HPF at 25 °C for 15 min. Then the roots were rinsed with distilled water three times, followed by visualization (excitation 490 nm and emission 515 nm) with a fluorescence microscope (ECLIPSE, TE2000-S, Nikon, Melville, NY, USA). The relative fluorescent density of HPF was analyzed using Image-Pro Plus 6.0 (Media Cybernetics, Inc., Rockville, MD, USA).

### 4.4. Histochemical Detection of Intracellular O_2_^•^¯ in Roots

Intracellular O_2_^•^¯ in roots was visualized in situ using specific fluorescent probe DHE described by Yamamoto et al. [70]. The roots of seedlings after treatment were incubated in 15 μM of DHE at 25 °C for 15 min. Then the roots were rinsed with distilled water three times, followed by visualization (excitation 535 nm and emission 610 nm) with a fluorescence microscope (ECLIPSE, TE2000-S, Nikon, Melville, NY, USA). The relative fluorescent density of DHE was analyzed using Image-Pro Plus 6.0 (Media Cybernetics, Inc., Rockville, MD, USA).

### 4.5. Histochemical Detection of Lipid Peroxidation in Roots

Histochemical detection of lipid peroxidation was achieved by using Schiff’s regent as described by Wang and Yang [71]. The roots of seedlings after treatment were incubated in Schiff’s reagent for 20 min. Then the stained roots were rinsed with a solution containing 0.5% (*w/v*) K_2_S_2_O_5_ (prepared in 0.05 M of HCl) until the root color became light red. After that, the roots were photographed by using a stereoscopic microscope (SteREO Discovery.V8, ZEISS, Oberkochen, Germany).

### 4.6. Histochemical Detection of Loss of Membrane Integrity in Roots

Histochemical detection of loss of plasma membrane integrity in roots was performed by using Evans blue as described by Yamamoto et al. [72]. The roots of seedlings after treatment were incubated in Evans blue solution (0.025%, *w/v*) for 20 min. After that, the roots were rinsed with distilled water three times, followed by imaging by using a stereoscopic microscope (SteREO Discovery.V8, ZEISS, Oberkochen, Germany).

### 4.7. Determination of TBARS Content in Roots

The concentration of TBARS was determined as an indicator of the level of lipid peroxidation in plants. A TBARS detection kit (A003; Nanjing Jiancheng Bioengineering Institute, Nanjing, China) was selected to measure the TBARS level based on the spectrophotometric determination of the reaction between TBARS and TBA (1,3-diethyl-2-thiobarbituric acid) assisted by TCA (trichloroacetic acid) [73].

### 4.8. Histochemical Detection of Intracellular H_2_S in Roots

The intracellular H_2_S in roots was visualized using specific fluorescent probe WSP-1 according to Li et al. [74] and Liu et al. [75]. The roots of seedlings after treatments were incubated at 20 mM Hepes-NaOH (pH 7.5) buffer solution containing 20 μM of WSP-1 at 25 °C for 40 min. Then the roots were rinsed with distilled water three times, followed by visualization (excitation 465 nm and emission 515 nm) with a fluorescence microscope (ECLIPSE, TE2000-S, Nikon, Melville, NY, USA). The relative fluorescent density of WSP-1 was analyzed using Image-Pro Plus 6.0 (Media Cybernetics, Inc., Rockville, MD, USA).

### 4.9. Histochemical Detection of Cell Death in Roots

Histochemical detection of cell death in roots was performed by using fluorescent probe PI (propidium iodide) in situ as described by Kellermeier et al. [76]. The roots of seedlings after treatment were incubated in 20 μM of PI solution for 20 min. Then the roots were rinsed with distilled water three times, followed by visualization (excitation 535 nm and emission 615 nm) with a fluorescence microscope (ECLIPSE, TE2000-S, Nikon, Melville, NY, USA). The relative fluorescent density of PI was analyzed using Image-Pro Plus 6.0 (Media Cybernetics, Inc., Rockville, MD, USA).

### 4.10. Histochemical Detection of Free Cd^2+^ in Roots

Free Cd^2+^ in roots was detected in vivo by using fluorescent probe Leadmium^TM^ Green AM [77]. The seedlings roots after treatment were harvested and washed in 1 mM of EDTA for 3 min, and rinsed with distilled water three times. Then the roots were incubated in 1 μg/mL of Leadmium^TM^ Green AM at 25 °C for 20 min. Then the roots were rinsed with distilled water three times, followed by visualization (excitation 488 nm and emission 525 nm) with a fluorescence microscope (ECLIPSE, TE2000-S, Nikon, Melville, NY, USA). The relative fluorescent density of Leadmium Green was analyzed using Image-Pro Plus 6.0 (Media Cybernetics, Inc., Rockville, MD, USA).

### 4.11. Analysis of Gene Expression

Real-time quantitative RT-PCR (reverse transcription-polymerase chain reaction) (qRT-PCR) was selected to quantify the expression levels of the genes. The sequences of *LCD* (XM_016597688.1) and *DCD* (XM_016611291.1) were obtained from NCBI (The National Center for Biotechnology Information) for the design of primers. Total RNA was extracted from roots using Trizol (Invitrogen) according to the manufacturer’s instructions. Reverse transcription was performed at 42 °C in 25 μL reaction mixture including 3 μg of RNA, 0.5 μg of oligo (dT) primers, 12.5 nmol of dNTPs, 20 units of RANase inhibitor and 200 units of M-MLV. The first cDNA was used as a template for real-time RT-PCR analysis (Applied Biosystems 7500 Fast Real-Time PCR System, Applied Biosystems, Waltham, MA, USA) with SYBR Premix Ex Taq^TM^ (TaKaRa Bio Inc., Dalian, China) according to the manufacturer’s instructions. The qPCR procedure was as follows: initial denaturation at 95 °C for 30 s, followed by 40 cycles of 95 °C for 5 s, 60 °C for 30 s, and 72 °C for 30 s. Data were collected and analyzed by using ABI 7500 software (v. 2.0.6, Applied Biosystems) based on 2^−ΔΔ*C*T^ threshold cycle method [78]. The relative abundance of *EF1-α* (*elongation factor 1-α*) was determined and used as the internal standard to normalize the data. The expression levels of corresponding genes are presented as values relative to the control samples under the indicated conditions. The primers used for amplifying the target genes are as follows: *LCD*, forward 5′-GGAGCCATGCTCTAGTGTTAAG-3′ and reverse 5′-CCAATCCTACTCCAGTGTTTCC-3′; *DCD*, forward 5′-GGTGGCCTACTGGGTTTATATG-3′ and reverse 5′-GCCGTCTTGTCTTGGGATAG-3′; *EF1-α*, forward 5′-ATGATGACGACGATGATGATA-3′ and reverse 5′-GTAAGCCCTTCTTGCTGAACAC-3′).

### 4.12. Cluster Analysis

Hierarchical cluster analysis for different parameters was performed by using Cluster 3.0 (http://bonsai.hgc.jp/~mdehoon/software/cluster/) [79]. All the data of different treatments were transformed and presented as relative fold change (log_2_) respect to their controls, respectively. The generated tree figure was displayed by using Java Treeview (https://sourceforge.net/projects/jtreeview/) [80].

### 4.13. Data Analysis

Each result was presented as the mean ± standard deviation (S.D.) of at least three replicated measurements. The significant differences between treatments were statistically evaluated by SD and one-way analysis of variance (ANOVA) using SPSS 14.0 (Statistical Package for the Social Science, SPSS Inc., Chicago, IL, USA). The data between two specific different treatments were compared statistically by ANOVA, followed by *F*-test if the ANOVA result is significant at *p* < 0.05. For multiple comparison analysis, least significant difference test (LSD) was performed on all data following ANOVA tests to test for significant (*p* < 0.05) differences among different treatments.
